# *SIMLIN*: a bioinformatics tool for prediction of S-sulphenylation in the human proteome based on multi-stage ensemble-learning models

**DOI:** 10.1186/s12859-019-3178-6

**Published:** 2019-11-21

**Authors:** Xiaochuan Wang, Chen Li, Fuyi Li, Varun S. Sharma, Jiangning Song, Geoffrey I. Webb

**Affiliations:** 1 Monash Centre for Data Science, Faculty of Information Technology, Monash University, Melbourne, VIC 3800 Australia; 2 Division of Cancer Epidemiology, Cancer Council Victoria, Melbourne, VIC 3004 Australia; 3 Institute of Molecular Systems Biology, Department of Biology, ETH Zürich, 8093 Zürich, Switzerland; 4 Infection and Immunity Program, Biomedicine Discovery Institute and Department of Biochemistry and Molecular Biology, Monash University, Melbourne, VIC 3800 Australia; 5 ARC Centre of Excellence for Advanced Molecular Imaging, Monash University, Melbourne, VIC 3800 Australia

**Keywords:** Protein post-translational modification, S-sulphenylation, Bioinformatics software, Machine learning, Ensemble learning

## Abstract

**Background:**

S-sulphenylation is a ubiquitous protein post-translational modification (PTM) where an S-hydroxyl (−SOH) bond is formed via the reversible oxidation on the Sulfhydryl group of cysteine (C). Recent experimental studies have revealed that S-sulphenylation plays critical roles in many biological functions, such as protein regulation and cell signaling. State-of-the-art bioinformatic advances have facilitated high-throughput in silico screening of protein S-sulphenylation sites, thereby significantly reducing the time and labour costs traditionally required for the experimental investigation of S-sulphenylation.

**Results:**

In this study, we have proposed a novel hybrid computational framework, termed *SIMLIN*, for accurate prediction of protein S-sulphenylation sites using a multi-stage neural-network based ensemble-learning model integrating both protein sequence derived and protein structural features. Benchmarking experiments against the current state-of-the-art predictors for S-sulphenylation demonstrated that *SIMLIN* delivered competitive prediction performance. The empirical studies on the independent testing dataset demonstrated that *SIMLIN* achieved 88.0% prediction accuracy and an AUC score of 0.82, which outperforms currently existing methods.

**Conclusions:**

In summary, *SIMLIN* predicts human S-sulphenylation sites with high accuracy thereby facilitating biological hypothesis generation and experimental validation. The web server, datasets, and online instructions are freely available at http://simlin.erc.monash.edu/ for academic purposes.

## Background

Post-translational modifications (PTMs) of the cellular proteome provide a dynamic regulatory landscape that include both rapid reversible modifications and long-lasting irreversible modifications to cellular perturbations [1]. In particular, reactive oxygen species (ROS), which are highly reactive and toxic molecules generated during mitochondrial metabolism, have been shown to play important signalling roles in the presence of oxidative stress and cellular pathophysiology in various complex diseases when their levels are altered in periods of cellular stress [2–5]. In the redox environment, S-sulphenylation (i.e. S-sulfenylation), a type of PTM that occurs at cysteine residues, is a fleeting and reversible covalent oxidation of cysteinyl thiols (Cys-SH) towards supheric acids (Cys-SOH) in the presence of hydrogen peroxide, which thereby acts as a rapid sensor of oxidative stress [6–12]. Thus far, a number of experiments have validated that S-sulphenylation plays important roles in regulating protein functions under both physiologic and oxidatively stressed conditions [7, 9–11, 13–19]. Despite the lack of knowledge regarding the specific functionality of this redox modification in human cell systems, it has been reported that S-sulphenylation is involved in many signal transduction processes, such as the deubiquitinase activity in ovarian tumors and growth factor stimulation [11, 17, 20]. Furthermore, including S-sulphenylation, more than 200 sulfenic modifications that have been identified in various situations, such as transcription factors, signaling proteins, metabolic enzymes, proteostasis regulators, and cytoskeletal components [17]. Although only approximately 2% of proteins in the human, mouse, and rat proteomes contain cysteine residues [21], it is essential to understand the underlying mechanisms that contribute to the residues’ critical roles in various biological processes, such as S-sulphenylation, regulation of oxidative PTMs, and the quantification of sulfenic modification processes [6, 7, 9, 10, 14–16].

Despite the significant progress in selective labelling methods for S-sulphenylation using β-dicarbonyl compounds dimedone and analogues, it remains challenging to accurately characterize protein S-sulphenylation sites experimentally, due to their intrinsic instability and low abundance of cysteine residues [6–8, 11, 17, 20, 22]. Moreover, experimental identification of S-sulphenylation is labour-intensive and particularly difficult due to its intrinsically unstable nature and the diversity of the redox reaction [7, 8, 11]. Therefore, in order to assist biologists with characterization of S-sulphenylation sites and S-sulphenylated sequences, it is imperative to construct a generalizable computational tool for highly accurate prediction of protein S-sulphenylation sites.

To date, several algorithms for S-sulphenylation prediction have been published, including MDD-SOH, SOHSite [6, 7], SOHPRED [23], Press [24], iSulf-Cys [25], SulCysSite [26], PredSCO [27], the predictor by Lei et al [28], and SVM-SulfoSite [29]. Among these computational tools, to the best of our knowledge, the most representative algorithm for S-sulphenylation prediction is MDD-SOH, along which the training dataset in this study was assembled. MDD-SOH is a two-stage ensemble learning model based only on SVM classifiers built upon the previous “SOHSite” project [6, 7]. Despite the progress of computational methods for S-sulphenylation prediction, the prediction performance needs to be further improved, due to the low abundance of cysteine residues and the insufficient number of experimentally verified S-sulphenylation sites.

In this study, we propose a novel bioinformatics tool for improved prediction of protein S-sulphenylation sites, named *SIMLIN*, integrating a number of protein sequence-derived and protein structural features based on the sequence motifs previously identified in [6, 7]. *SIMLIN* is a two-layer framework consisting of Support Vector Machine (SVM) and Random Forests (RF) in the first layer and neural network models in the second layer. To further improve the prediction accuracy of *SIMLIN*, an incremental feature selection method was employed, based on by the mRMR approach implemented in the R package “mRMRe” [30]. The constructed SVM and RF models, trained on different feature clusters plus the selected feature set, were used as the input for the neural network in the second layer. Empirical assessment on the independent testing dataset demonstrated that *SIMLIN* achieved a prediction accuracy of 88% and an AUC score of 0.82, outperforming the existing methods for S-sulphenylation site prediction.

## Implementation

**Fig. 1:**
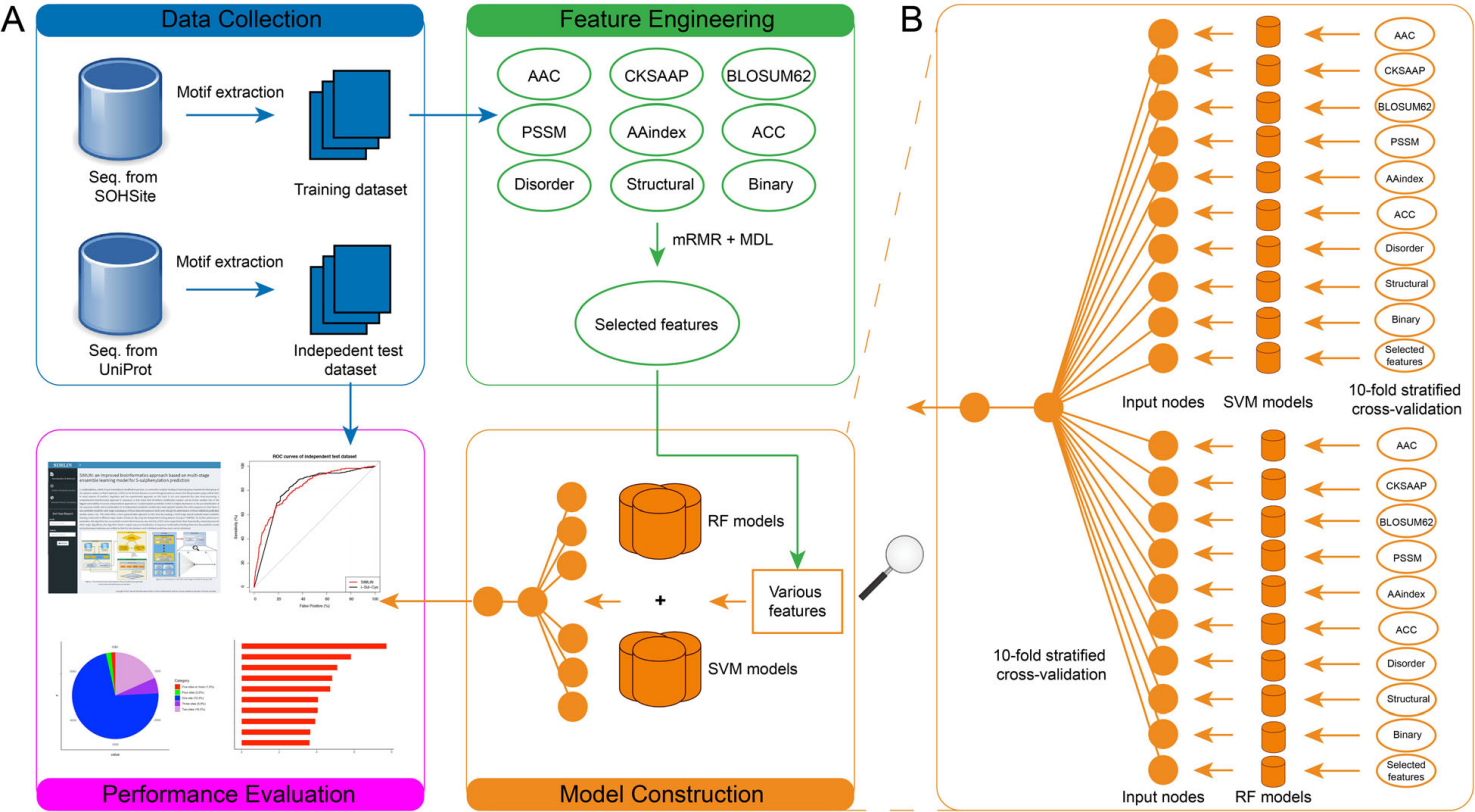
The overall framework illustrating the model construction and performance evaluation for *SIMLIN*. **a** The four major steps for constructing *SIMILIN* include data collection, feature engineering, model construction, and performance evaluation, (**b**) A detailed breakdown of the construction of the two-stage hybrid *SIMLIN* model

Figure 1 provides an overview of the framework of *SIMLIN*, which consists of four major steps: (i) data collection, (ii) feature calculation and selection, (iii) model training, and (iv) performance evaluation. During the data collection process, we collected experimentally verified S-sulphenylation sites from the study of Bui et al. [7]. The negative dataset (defined as proteins without experimentally validated S-sulphenylation sites) was extracted from the UniProt database [31]. Refer to the section 2.1 for more details regarding data collection and pre-processing. For feature extraction, a variety of protein sequence and structural features were extracted and selected using the MDL (minimum descriptive length) technique [32] and mRMR (minimum-redundancy maximum-relevancy) algorithm [30, 33]. A detailed description and statistical summary of the calculated features are provided in the Section 2.2. To construct accurate predictive models, at the ‘Model Construction’ step, a generalized ensemble framework of *SIMLIN* was developed by integrating various machine-learning algorithms including Artificial Neural Networks (ANNs) [34, 35], SVMs with various kernel functions [36, 37], and RFs [38]. To evaluate and compare the prediction performance of *SIMLIN* with the existing methods, at the last step, we assessed the prediction performance of different algorithms on both 10-fold stratified cross-validation sets and independent datasets assembled in the previous study of Bui et al [7].

### Data collection and pre-processing

Both benchmark and independent test datasets in this study were extracted from the ‘SOHSite’ web server, constructed by Bui et al. [6, 7]. Sequence redundancy of the dataset was removed in this study (using 30% as the sequence identity threshold), which was reported to be the most complete dataset for S-sulphenylation to date through the integration of experimentally validated S-sulphenylation sites from four different resources: (i) the human S-sulphenylation dataset assembled using a chemoproteomic workflow involving the S-sulfenyl-mediated redox regulation [11], by which the S-sulphenylation cysteines were identified; (ii) the RedoxDB database [39], which curates the protein oxidative modifications including S-sulphenylation sites; (iii) the UniProt database [31], and (iv) related literature. Considering the frequent updates of UniProt, based on the gene names provided in the datasets, we further mapped these proteins to the UniProt database (downloaded November 2016). The canonical protein sequences harboring experimentally verified S-sulphenylation sites were retrieved and downloaded from the UniProt database. Motifs of 21 amino acids with the S-sulphenylation site in the center and flanked by 10 amino acids each side were then extracted from the protein sequences. The highly homologous motifs have been further removed to maximize the sequence diversity according to [7, 13]. The resulting dataset contains a total of 1235 positive samples (i.e. with S-sulphenylation sites) and 9349 negative samples (i.e. without S-sulphenylation sites). Table 1 provides a statistical summary of the benchmark and independent test datasets, respectively.

**Table 1:** The statistics of datasets employed in this study

	Number of positive motifs	Number of negative motifs	Total
Training dataset	1019	7937	8956
Independent test dataset	216	1412	1628
Total	1235	9349	10,584

### Feature extraction and calculation

To numerically represent the sequence motifs in the datasets, we calculated and extracted both sequence-based and structural features [40]. In total nine types of sequence-derived and structural features were extracted and used, including the composition of *k*-spaced amino acid pairs (CKSAAP) [41], motif binary representations [42], amino acid substitution matrix (BLOSUM62) [43], protein specific scoring matrix (PSSM) by PSI-BLAST [44], amino acid index (AAindex) [45], amino acid composition (AAC), surface accessibility (ACC) based on protein secondary structure prediction, protein predicted disordered region, and protein predicted secondary structure. The detailed information about each type of features and its feature dimensionality is shown in Table 2.

**Table 2:** The sequence and structural features extracted and the feature dimensionalities

Feature type	Feature Cluster	Dimension
Sequence	AAC	20
	CKSAAP	2400
	BLOSUM62	441
	PSSM	400
	AAindex	1344
	Binary	441
Structural	Predicted protein disordered region	20
	Predicted protein secondary structure	84
	Predicted surface accessibility	147
Total		5297

#### Composition of k-spaced amino acid pairs (CKSAAP)

The CKSAAP encoding theme has been widely applied [46–49], which represents a protein sequence using the compositions of amino acid pairs spaced by the *k* residues [41, 50, 51]. The composition of each possible *k*-spaced amino acid pair *i* can be therefore calculated based on the following formula:

1 $$ CKSAAP\left[i=1,2,3,\dots \left({k}_{max}+1\right)\times 400\right]={N}_i/\left(W-k-1\right), $$

where *N*_*i*_ is the number of the *k*-spaced amino acid pair *i*, *W* denotes the window size, and *k*_*max*_ represents the maximum space considered — which has been optimized as *k*_*max*_ = 5 in this study [42]. In total, the CKSAAP scheme generated a feature vector of 2400 dimensions for each motif.

#### Motif one-hot encoding (binary)

Each motif was also presented using a binary encoding scheme [42], where each amino acid in the motif was denoted using a 21-dimensional vector organized via the alphabetic order of 20 natural amino acids and a gap-filling residue “X”. The value 1 was used to denote that the amino acid was in fact in the motif and was placed in its corresponding position in the vector, while other positions in the vector were filled with “0”. For instance, the residue C (cysteine) is denoted as {0,1,0,0,0,0,0,0,0,0,0,0,0,0,0,0,0,0,0,0,0}. Therefore, for a motif with 21 amino acids, a total of 441 (21 × 21) features were generated using the motif binary representation scheme.

#### Amino acid substitution matrix (BLOSUM62)

The BLOSUM62 is a widely used amino acid substitution matrix based on sequence alignment [43, 52] and has been employed in a variety of bioinformatic studies [6, 22, 53–55]. For each amino acid, a 21-dimensional vector consisting of substitution scores of all 20 amino acids and an additional terminal signal constitute the matrix. For each motif, a 21 × 21 matrix was used and a total number of 441 features were added.

#### Position-specific scoring matrix (PSSM)

Using the UniRef90 dataset from the UniProt database, we performed PSI-BLAST (version 2.2.26) search to generate the PSSM for each motif in our dataset to represent the sequence conservation and similarity scores. PSSM has been widely applied in a variety of bioinformatics studies as a crucial sequence feature type. Similar to the feature representation of BLOSUM62, 441 features were finally generated for each motif.

#### Amino acid index (AAindex)

AAindex is a collective database that provides a variety of physical and chemical properties of amino acids [45]. A number of bioinformatics studies have benefited from use of these amino acid properties provided in the AAindex database [46, 48, 56]. Due to the high diversity of the properties offered in the AAindex database, Saha et al. [57] further categorized these indices into eight clusters, which were used for the AAindex feature set for each motif in our study. Therefore, we utilized a selected set of AAindex (i.e., a vector of 1344 dimensions (21 × 8 × 8) [52] attributes to represent each motif.

#### Amino acid composition (AAC)

For the ACC encoding, each motif is represented as a 20-dimensional vector, where each dimension denotes the number of occurrence of each amino acid within the given motif and is further normalized (i.e. divided by the length of the motif [22]).

#### Predicted protein disordered region

Given the strong relationships between protein disordered regions and PTMs [58–63], we also integrated the predicted disordered region of a protein as a feature set. To do so, we conducted protein disordered region prediction using DISOPRED (Version 3.1) [64] based on protein sequence. Each amino acid is given a predictive score by DISOPRED, which indicates the likelihood of being located in the protein’s disordered region. For a sequence motif of 21 residues, a 20-dimensional vector of predicted scores (i.e. 10 scores for the upstream and 10 scores for the downstream amino acids, respectively) was constructed.

#### Predicted protein secondary structure

PSIPRED (Version 3.5) [65, 66] was employed to predict protein secondary structure based on the protein’s amino acid sequence. The predictive outputs of PSIPRED contain four scores for each residue including the predicted structural class (i.e. C, coil; E, beta strand; and H, alpha helix) and the probabilities of each structural class. As a result, for a motif with 21 amino acids, an 84-dimensional (including three probabilities and the recommendation for each residue) vector was generated for the predicted protein secondary structure feature.

#### Predicted surface accessibility (ACC)

The surface accessibility feature was calculated using the NetSurfP-1.1 algorithm [67] based on the protein sequences. Each residue in the protein is represented using seven predictive scores, indicating the accessibility (i.e. if this residue is buried), relative surface accessibility, absolute surface accessibility, Z-fit score, probability of this residue being in alpha-helices, beta-strands, and coils. Note that the predictive scores of each category generated by NetSurfP range widely. Therefore, we employed the Min-Max method to normalize the prediction scores of each type [35]. The formula we used for the data normalization was as follows:

2 $$ {\mathrm{V}}_{ij}=\frac{{\mathrm{V}}_{ij}-{\mathit{\min}}_{j\in \left\{1\dots m\right\}}\left\{{\mathrm{V}}_{ij}\right\}}{{\mathit{\max}}_{j\in \left\{1\dots m\right\}}\left\{{\mathrm{V}}_{ij}\right\}-{\mathit{\min}}_{j\in \left\{1\dots m\right\}}\left\{{\mathrm{V}}_{ij}\right\}}, $$

where *V*_*ij*_ represents the value *i* of the feature category vector *j*, and *m* denotes the number of observations represented in the vector *j*. As a result, all values were rescaled to the range between 0 and 1.

### Feature selection

As shown in Table 2, a total of 5297 sequence and structural features were calculated and extracted. Such high-dimensional feature vectors might contain misleading and noisy information, which would lead to biased model training. Furthermore, it would require considerable time and effort to build computational models based on such high-dimensional feature set. Therefore, we employed the mRMR (minimum Redundancy Maximum Relevance) [30, 33] package and forward incremental feature selection to eliminate noisy and less informative features from the original feature vector. To perform feature selection, we first applied mRMR to calculate and rank the importance score of each feature. Then, based on the feature importance ranking provided by mRMR, we initiated an empty set and added one feature from the original feature set at a time. The AUC values based on the current feature set were evaluated for both RF and SVM independently, and the resulting feature subset was formed using the features that resulted in higher AUC values for both SVM and RF models. Each feature was incrementally added into the optimized feature set based on the scores of feature importance provided by the mRMR until the curve of AUC values achieved its peak. As described, by applying this forward stepwise sequential variable elimination, the feature with the highest importance was selected. According to the RF algorithm, the global permuted importance is based on the out-of-bag sample *B* of the tree *t* in the forest *F* for each feature *X*_*j*_ and is defined as follows [22, 35, 38]:

3 $$ {f}_{imp}\left({X}_j\right)=\frac{\sum_{i\in B}I\left({y}_i={y}_i^{\prime}\right)-I\left({y}_i={y}_{ij}^{\prime}\right)}{\mid B\mid }. $$

### Model construction

As shown in Fig. 1, the development of *SIMLIN* consists of two major stages after feature selection: (i) employing SVM and RF models based on different feature types (Table 2) to generate the input for the neural network models, and (ii) training of the neural network model based on the optimized RF and SVM models to deliver the final predictive outputs. During the first stage, ten RF and SVM models were constructed based on the nine types of features and the selected feature set. 10-fold stratified cross-validation was performed on the training dataset to select the best model (i.e. with highest AUC values) for each feature type. During the second stage, we built a neural network model which consists of three layers including an input layer, a hidden layer, and an output layer. The first layer harbours 20 nodes to take the output of the best RF and SVM models as the input based on the 10-fold stratified cross-validation performed during the first stage, while the hidden and output layers only have one node (denoted as *H*_*1*_ and *O*_*1*_, respectively). Furthermore, in the hidden layer, in addition to *H*_*1*_, two extra nodes, *B*_*1*_ and *B*_*2*_, were auto-generated nodes by the neural network algorithm for the purpose of model balancing. Lastly, the *O*_*1*_ node in the output layer represents the prediction outcome from the entire algorithm.

We applied a number of software packages to implement *SIMLIN* in our study, including the Python-based machine learning package “scikit-learn” [68], and various R packages of SVM (combining “kernelab” and "e1071") and neural network model (“nnet”) [35, 69]. The feature selection techniques employed in our study, including mRMR and MDL, were implemented based on the R packages “mRMRe” and “discretization” [70–72], respectively. Additionally, R packages “caret” [73] and “fscaret” [74] have been used in combination for the control of overall workflow for model training and parameter optimization.

### Prediction performance evaluation

We applied widely used measures to evaluate and compare the prediction performance of *SIMLIN*, including the Area Under the Curve (AUC), Accuracy, Sensitivity, Specificity and Matthew’s Correlation Coefficient (MCC) [75–77]. During the model training process, AUC was used as the main measure for parameter optimization. The performance measures used are defined as follows:

$$ Accuracy=\frac{TP+ TN}{TP+ FP+ TN+ FN}, $$

$$ Sensitivity=\frac{TP}{TP+ FN}, $$

$$ Specificity=\frac{TN}{TN+ FP}, $$

$$ MCC=\frac{TP\times TN- FP\times FN}{\sqrt{\left( TP+ FN\right)\times \left( TN+ FP\right)\times \left( TP+ FP\right)\times \left( TN+ FN\right)}}, $$

where *TP*, *TN*, *FP,* and *FN* denote the numbers of true positives, true negatives, false positives and false negatives, respectively. In this study, the S-sulphenylation sites were regarded as the positives, while the non-S-sulphenylation sites were considered as the negatives for the statistics of AUC, specificity and sensitivity.

## Results and discussion

### Motif conservation analysis and feature selection

**Fig. 2:**
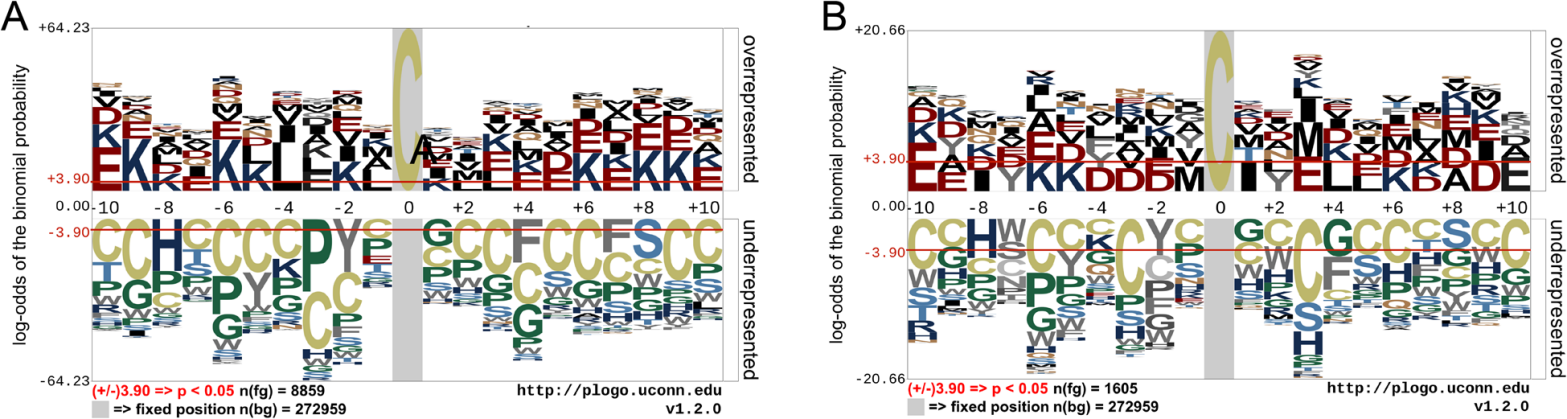
Motif conservation analysis of S-sulphenylation using the human proteome as the background set for (**a**) benchmarking and (**b**) independent datasets

We first performed the motif conservation analysis using both benchmarking and independent test datasets. Two sequence logos with the human proteome as the background set generated by pLogo are shown in Fig. 2. In general, the over- and under-represented amino acids surrounding the central cysteine are similar across the benchmarking and independent test datasets. In accordance with the conclusion by Biu et al., amino acids such as leucine (L), lysine (K), glutamate (E), and aspartate (D) are over-represented, while cysteine (C), serine (S), and phenylalanine (F) are under-represented.

Prior to the construction of *SIMLIN*, based on the calculated and extracted features (Table 2), we generated another feature set which contains selected features from the original combined features (i.e. AAC, CKSAAP, BLOSUM62, PSSM, AAindex, ACC, Protein predicted disordered region, Protein secondary structure prediction, and Binary) using stepwise forward sequential variable elimination. As a result, the AUC achieved its highest value of 0.72 (sensitivity: 0.95; specificity: 0.19; accuracy: 86.6%; MCC: 0.182) when 166 features were selected. Among the selected 166 features, 110 (66.3%) and 56 (33.7%) were sequence and structural features, respectively. A detailed breakdown list of these features in terms of feature types and names is available in supplementary material (Additional file 1: Table S1).

### Model constructions in the two stages of *SIMILN*

**Fig. 3:**
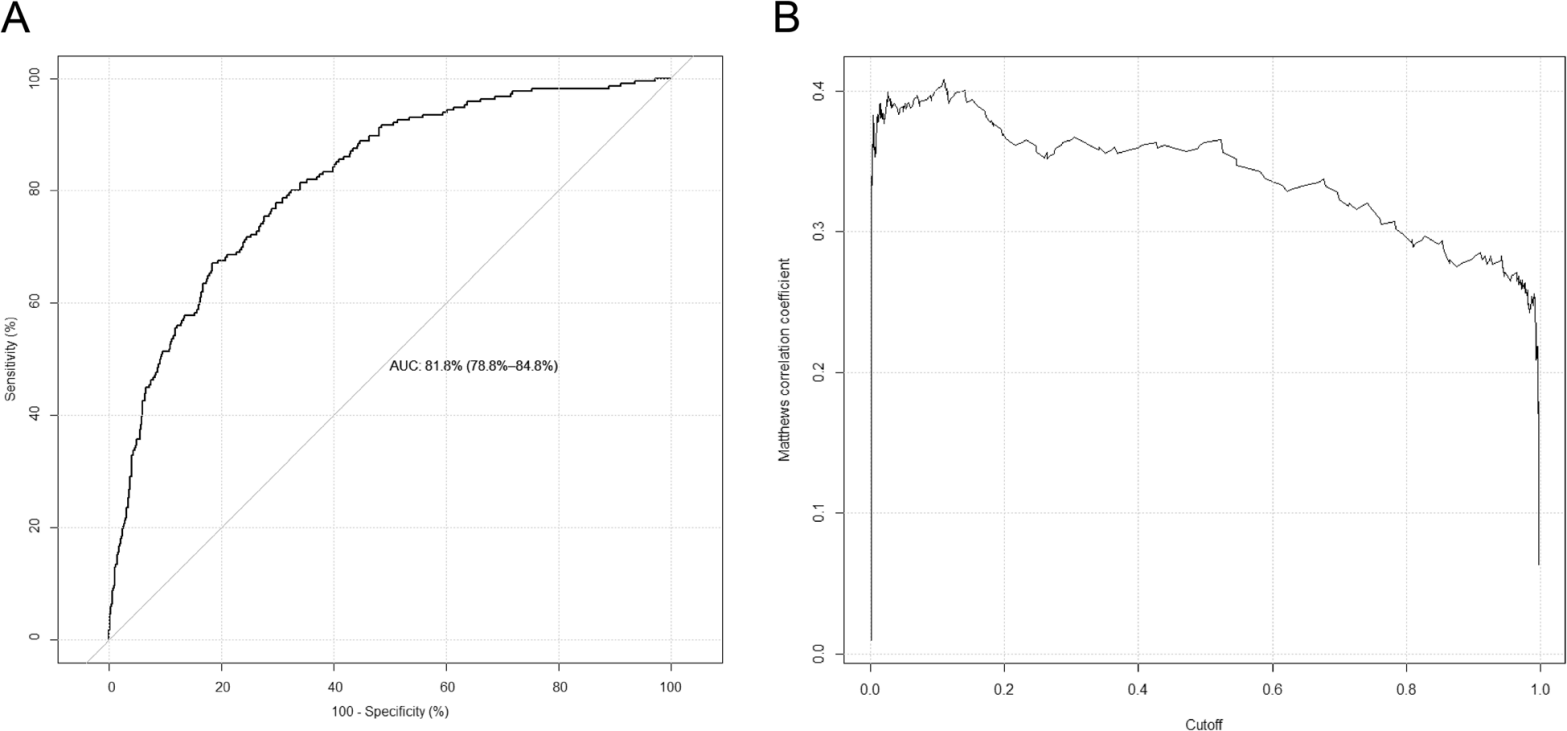
Prediction performance of *SIMLIN* on the independent test dataset in terms of (**a**) ROC and (**b**) MCC

At the first stage of *SIMILN* construction, we built nine SVM and RF models based on the nine clusters of calculated features (Table 2), respectively. Additionally one SVM and RF models were also constructed using the set of selected features (Additional file 1: Table S1). The RF and SVM models were constructed and assessed via 10-fold stratified cross-validation and the average AUC values are shown in Table 3. For the RF models, to reach the optimal performance, the number of trees was set to the nearest integer of the subspace dimensionality of the classification task, which is the square root of the predictors’ number. For the SVM models, different kernels were used including the polynomial, radial sigma, and linear kernels for each feature set. The AUC-based performance optimization and kernel selection was performed automatically by the R packages “caret” and “kernelab”. The best-performing kernels and their corresponding AUC values were listed in Table 3. It can be seen from Table 3 that SVM and RF models provided competitive performance when using different types of features; however, the RF model outperformed the SVM model on the selected feature set. As shown in Fig. 3, the outputs of the 20 constructed models (i.e. ten RF and ten SVM models; the first layer) were used as inputs for the second layer, i.e. the neural network model, where the nodes, from *I*_*1*_ to *I*_*20*_ took the output of the 20 models based on the outputs of RF and SVM models.

**Table 3:** The AUC values of RF and SVM models constructed using different feature sets at the first stage

Feature sets	AUC	
	RF (class weight balanced)	SVM (kernel function)
AAC	**0.68**	0.63 (Polynomial kernel)
AAindex	0.69	0.69 (Radial basis function kernel with grid search hyperparameter tuning)
ACC	**0.71**	0.64 (Radial basis function kernel)
BINARY	0.59	**0.71** (Polynomial kernel)
BLOSUM62	0.68	**0.74** (Radial basis function kernel)
CKSAAP	**0.66**	0.63 (Polynomial kernel)
DISOPRED	0.54	**0.55** (Linear kernel)
PSIPRED	**0.62**	0.60 (Polynomial kernel)
PSSM	**0.73**	0.71 (Polynomial kernel)
Selected features (mRMR+forward consequential elimination)	**0.75**	0.72 (Linear kernel)

The bold font shows the highest performance of each feature among the RF and SVM

At the second stage a Feed-Forward Neural Network with three layers - including an input layer (20 nodes), a hidden layer (3 nodes) and an output layer (1 node) — was constructed using the R package ‘nnet’ and subsequently evaluated. Similar to the RF and SVM construction, 10-fold stratified cross-validation was employed using the training dataset for building the neutral network model. During the training process, two parameters (i.e. the number of units in the hidden layer and the weight decay for optimising the performance and minimizing the overfitting) were automatically adjusted and evaluated by the network model. The values of the two parameters were adjusted automatically and the resulting performance including AUC, sensitivity, and specificity are given in Table 4. Generally, the performance achieved using different numbers of units in the hidden layer and weight decay values was satisfactory. Based on the performance, the number of units and the weight decay were set to 1 and 0.1 in the final neural network model, respectively (Additional file 1: Table S2). This was for the purpose of minimizing the number of nodes in the hidden layer while maximining the AUC value and convergence rate.

**Table 4:** Prediction performance of the neural network model with different units in the hidden layer via 10-fold stratified cross-validation test

#Units in the hidden layer	Decay	AUC	Sensitivity	Specificity
1	0	0.999842 ± 3.15E-4	0.999685 ± 6.30E-4	1
	0.0004	0.999994 ± 6.30E-5	0.999887 ± 3.62E-4	1
	0.1	1	0.999874 ± 3.68E-4	1
3	0	0.999874 ± 3.35E-4	0.999723 ± 6.84E-4	1
	0.0004	0.999987 ± 8.85E-5	0.999937 ± 2.76E-4	1
	0.1	1	0.999874 ± 3.80E-4	1
5	0	0.999793 ± 5.90E-4	0.999685 ± 7.02E-4	0.999902 ± 9.80E-4
	0.0004	0.999869 ± 7.28E-4	0.999912 ± 4.48E-4	0.999704 ± 2.20E-3
	0.1	1	0.999899 ± 3.44E-4	1

### Independent test and performance comparison with existing methods

We assessed and compared the prediction performance of *SIMLIN* with state-of-the-art methods for S-sulphenylation prediction on the independent test dataset. The compared approaches included MDD-SOH, SOHSite [6, 7], SOHPRED, PRESS, iSulf-Cys, SulCysSite. We also noticed that several new computational frameworks have been published recently, including PredSCO [27], the predictor by Lei et al [28], and SVM-SulfoSite [29]. However, due to the inaccessibility of source codes or implemented webservers, we were not able to compare their prediction results on our independent test dataset with the performance of *SIMLIN*. From Table 5 and Fig. 3, it is clear that generally *SIMLIN* outperformed the compared approaches. Compared to MDD-SOH, an important advantage of *SIMLIN* is that it does not require any pre-classified motifs. iSulf-Cys is another computational framework that employs a similar approach to create a unified predictive model, but it only used SVM models with three major encoding features (AAindex, binary and PSAAP) for model construction. The overall performance of iSulf-Cys is lower than *SIMLIN*. On the 95% CI the accuracy of iSulf-Cys is 0.7155 ± 0.0085; while *SIMLIN* achieved a prediction accuracy of 0.88 (0.857–0.892) on the 95% CI. The MCC value of *SIMLIN* was also higher than iSulf-Cys (0.39 vs. 0.3122). The SulCysSite model is mainly developed based on the multistage RFs with four major features (AAindex, binary amino acid codes, PSSM, and compositions of profile-based amino acids). Although SulCysSite achieved an AUC of 0.819, it used a biased approach whose final decision was dependent on a complex series of rules, each of which can only cover a small subset. In general, *SIMLIN* outperformed all the compared methods in terms of sensitivity, MCC, and AUC, demonstrating its ability to accurately predict human S- sulphenylation sites.

**Table 5:** Performance comparison with existing approaches for S-sulphenylation prediction on the independent test

Method	Sensitivity	Specificity	MCC	Accuracy	AUC
SOHPRED	0.73	0.74	0.34	N.A.^b^	0.80
PRESS	0.68	0.69	0.27	73.8%	N.A.
iSulf-Cys	0.73	0.64	0.31	66.8%	0.72
SulCysSite	0.77	0.71	N.A.	72.0%	0.76
*SIMLIN*	**0.88**	0.56	0.39	**88.0%**	0.82
MDD–SOH^a^	0.85	**0.87**	**0.58**	87.0%	N.A.

^a^The performance values of MDD-SOH were extracted from the study of Bui et al [6]

^b^N.A.: not available

The bold font shows the highest performance of each feature among the RF and SVM

### Proteome-wide prediction and functional enrichment analysis

In order to more effectively portray the distribution of predicted S-sulphenylation sites and their potential molecular functions, we performed human proteome-wide S-sulphenylation site prediction using the protein sequences collected from the UniProt database (Version Sep 2017) and our proposed *SIMLIN* framework. We first conducted statistical analysis on the distribution of predicted S-sulphenylation sites in proteins followed by a Gene Ontology (GO) enrichment analysis to reveal the potential cellular localization, biological function, and signalling/metabolic pathways involved in the predicted S-sulphenylation sites using the DAVID biological functional annotation tool (Version 6.8) [78, 79].

**Fig. 4:**
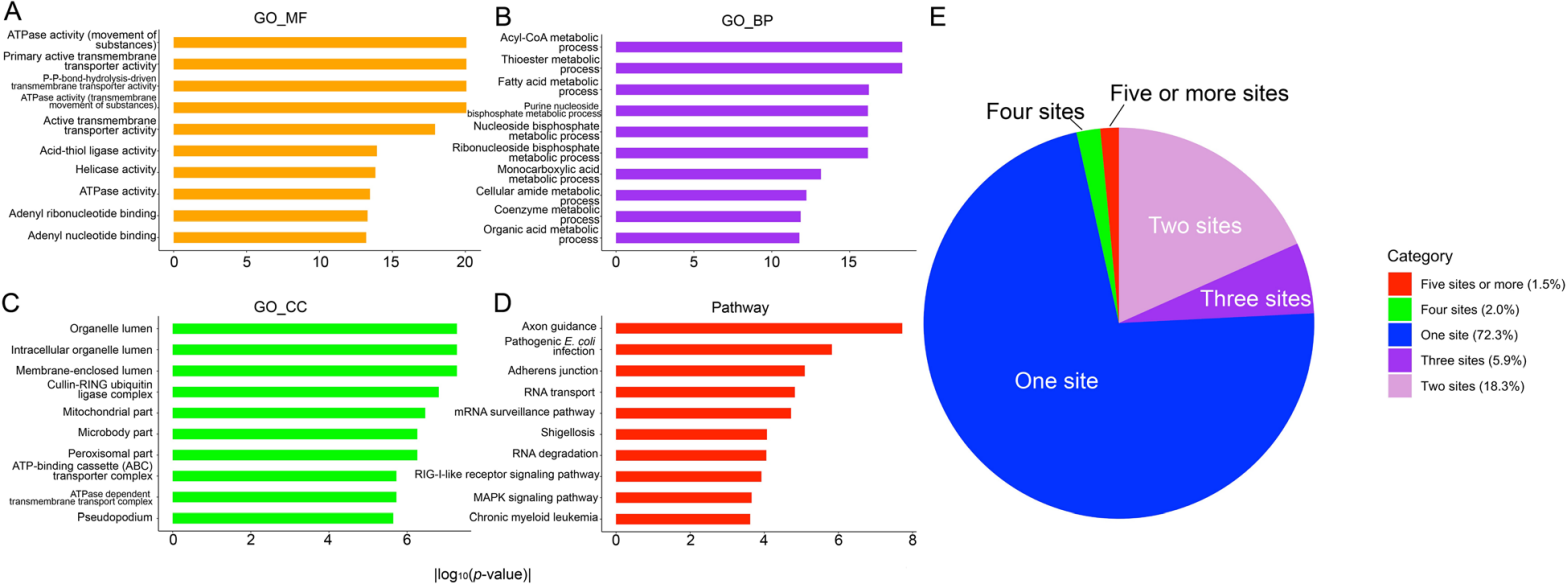
Gene ontology enrichment analysis of the predicted protein S-sulphenylation sites in the human proteome using *SIMLIN*: top 10 significant (**a**) molecular function terms (GO_MF), (**b**) biological process terms (GO_BP), (**c**) cellular component terms (GO_CC), (**d**) pathways; and (**e**) distribution of the numbers of predicted S-sulphenylation sites

Figure 4a-d display the top ten enriched candidates of our gene ontology and pathway enrichment analysis, in terms of molecular function, biological process and cellular component. Figure 4e shows the distribution of numbers of predicted S-sulphenylation sites in the human proteome. In terms of molecular function, the ATPase related activities (i.e., ATPase activity, coupled to movement of substances with a significant *p*-value of 8.5 × 10^− 21^; ATPase activity, coupled to transmembrane movement of substances - 8.5 × 10^− 21^; ATPase activity - 3.42 × 10^− 14^) have been found to be significantly enriched in proteins with predicted S-sulphenylation sites (Fig. 4a). An example of such relationship has been demonstrated in the study by Wojdyla et al. [80] where Acetaminophen (APAP) treatment has been shown to influence the ATP production, and the APAP-induced S-sulphenylation may act as one contributing fact to such effect. All enriched biological processes shown in Fig. 4b are metabolic processes, which indicate the important roles of S-sulphenylation in metabolism [11]. For instance, one S-sulphenylation occurring at C212 of a fatty acid synthase (FASN) protein may play a role in blocking an active site (C161), which is responsible for fatty acid synthase (Fig. 3B; fatty acid metabolic process - 5.82 × 10^− 17^) [11, 81]. While for cellular component category (Fig. 4c), the top three localisations are organelle (5.30 × 10^− 08^), intracellular organelle (5.30 × 10^− 08^) and membrane-enclosed lumens (5.30 × 10^− 08^), which is consistent with the analysis of Bui et al [6, 7] RNA transport is an important process associated with protein synthesis, which consists of 14 proteins enriched in S-sulphenylation and S-nitrosylation sites [80], highlighting the necessity of protein S-sulphenylation sites in RNA transport (Fig. 4d; 1.50 × 10^− 05^). Figure 3e shows the distribution of the numbers of predicted S-sulphenylation site contained in each protein. Expectedly, most of the proteins (72.3%) only contain one predicted site; while only 1.5% of the human proteome harbour five or more predicted sites. A full list of the predicted S-sulphenylation sites on human proteome is freely available on the *SIMLIN* webserver.

### Case study of predicted S-sulphenylation using *SIMLIN*

As aforementioned, compared with the dataset used for training *SIMLIN*, three more S-sulphenylation sites have been recently identified and added to the UniProt database, including BRF2_HUMAN (position 361 of Q9HAW0) [82], PTN7_HUMAN (position 361 of P35236; by similarity according to UniProt) and UCP1_HUMAN (position 254 of P25874; by similarity according to UniProt). *SIMLIN* precisely predicted all of these three S-sulphenylation sites, with the possibility scores of 0.997, 0.999 and 0.998, respectively, illustrating the predictive power and capacity of *SIMLIN* for predicting human S-sulphenylation sites.

### Implementation and usage of the *SIMLIN* webserver

**Fig. 5:**
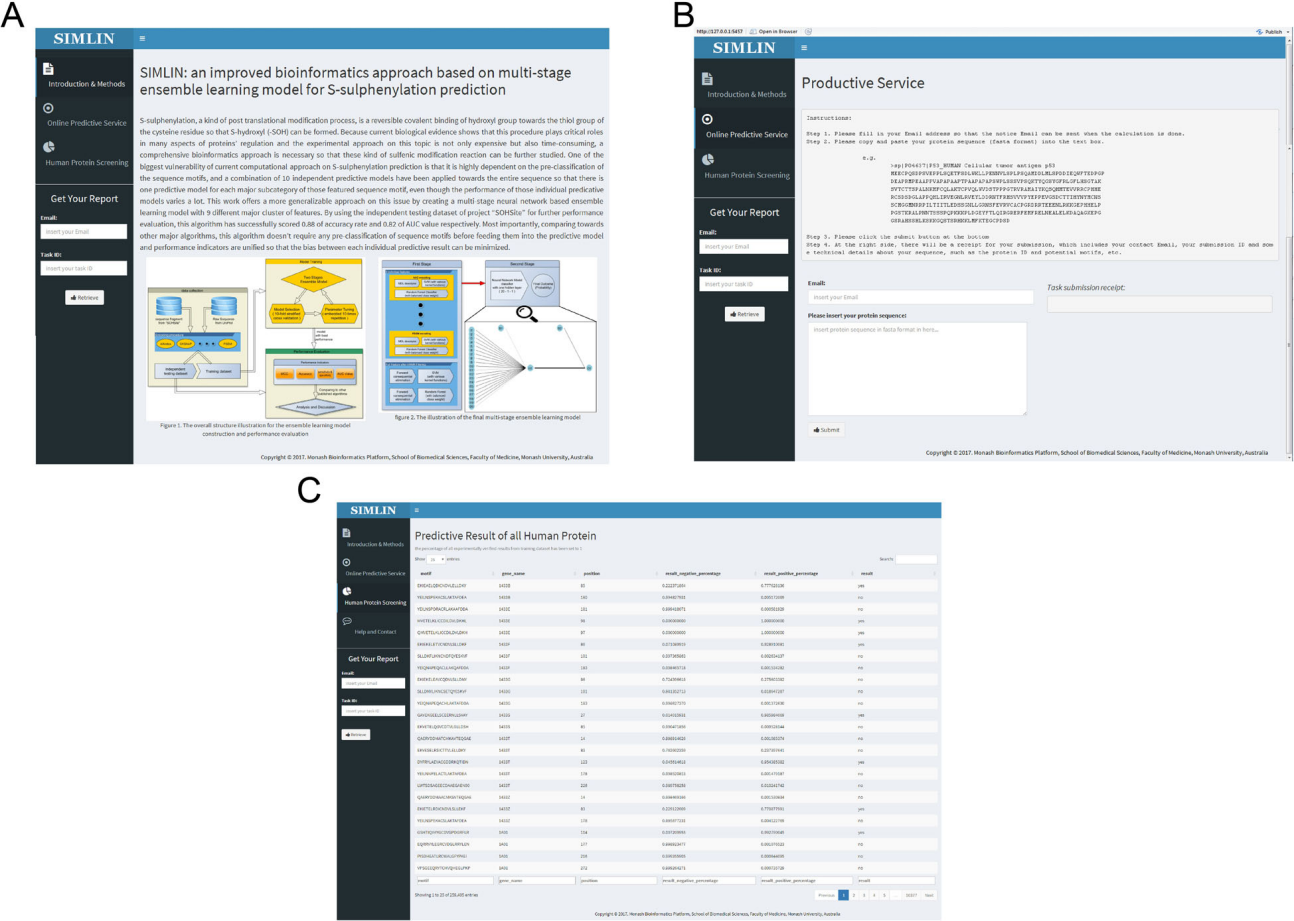
Screenshots of *SIMLIN* server (**a**) home page, (**b**) submission page, and (**c**) full list of the predicted S-sulphenylation sites of human proteome on the *SIMLIN* webserver

The open-access web application for *SIMLIN* was implemented using the Shiny framework (Version 1.3.0.403) in R language combining with Node.js (Version 0.10.21) and is freely available for academic use at http://simlin.erc.monash.edu/. The *SIMLIN* server resides on a Linux server, equipped with dual AMD Opteron CPUs, 8 GB memory, and 10 GB disk space. *SIMLIN* accepts both individual protein and a sequence file with the size limit of 1 MB as the input in FASTA format. An ‘Example’ link has been provided to demonstrate the predictive functionality of the service and guide users to conveniently use it. As the training dataset of *SIMLIN* was collected from the human proteome, the prediction results delivered by *SIMLIN* should be interpreted at the users’ discretion if the input protein is from other species rather than *Homo sapiens*. A graphical illustration of the *SIMLIN* webserver in terms of input and output is provided in Fig. 5.

## Conclusion

In light of the biological importance of S-sulphenylation, it is imperative to develop easy-to-use computational approaches for the accurate identification of S-sulphenylation sites. In this article, we present *SIMLIN*, a hybrid computation al framework integrating RF, SVM, and neural network models and sequence and structural features of S-sulphenylated motifs and proteins. Performance assessment on both cross-validation and independent test sets demonstrated that *SIMLIN* achieved outstanding prediction performance compared to state-of-the-art computational approaches (MDD-SOH, SOHSite, SOHPRED, PRESS, iSulf-Cys, and SulCysSite) for S-sulphenylation prediction. A user-friendly webserver has also been implemented to provide high-quality predictions of human S-sulphenylation sites using the optimised hybrid *SIMLIN* framework. Proteome-wide prediction of S-sulphenylation sites for the entire human proteome extracted from the UniProt database, has been made available at the *SIMLIN* webserver, aiming to provide highly accurate S-sulphenylation sites and facilitate biologists’ efforts for experimental validation, hypothesis generation, and data analysis. We anticipate that *SIMLIN* will be explored as a useful tool for human S-sulphenylation prediction. This effective framework can also be generally applied to address the prediction problem of other protein PTMs.

## Supplementary information

**Additional file 1: Table S1.** A detailed summary of the selected sequence and structural features using the MDL and mRMR feature selection methods. **Table S2.** The assigned weights of each node in the final neural network model.

## Data Availability

The datasets of this study are available at http://simlin.erc.monash.edu/.

## Abbreviations

AAC: amino acid composition
ACC: accuracy
ACC: surface accessibility
ANN: artificial neural network
AUC: area under the ROC curve
CKSAAP: composition of k-spaced amino acid pairs
FN: false negative
FP: false positive
GO: gene ontology
MCC: Matthews’ Correlation Coefficient
MDL: minimum descriptive length
mRMR: minimum Redundancy Maximum Relevance
PSSM: protein-specific scoring matrix
PTM: post-translational modification
RF: Random Forest
SVM: Support Vector Machine
TN: true negative
TP: true positive
