# Supplementary material for: SIMLIN: a bioinformatics tool for prediction of S-sulphenylation in the human proteome based on multi-stage ensemble-learning models
Source: BMC Bioinformatics. 2019 Nov 21;20:602. doi: 10.1186/s12859-019-3178-6 (PMC6868744; doi:10.1186/s12859-019-3178-6)
Supplement: Supplementary file 1 — Additional file 1: Table S1. A detailed summary of the selected sequence and structural features using the MDL and mRMR feature selection methods. Table S2. The assigned weights of each node in the final neural network model. [file 12859_2019_3178_MOESM1_ESM.docx]

Table S1. A detailed summary of the selected sequence and structural features using the MDL and mRMR feature selection methods

| **Feature Type** | **Feature Cluster** | **Number of Features** |
| --- | --- | --- |
| Sequence | AAC | 11 |
|  | AAindex | 15 |
|  | CKSAAP | 18 |
|  | BLOSUM62 | 3 |
|  | PSSM | 11 |
|  | Binary | 52 |
| **Subtotal:** | | **110 (66.3%)** |
| Structural | ACC | 49 |
|  | Predicted protein disordered region | 1 |
|  | Predicted Protein Secondary Structure | 6 |
| **Subtotal:** | | **56 (33.7%)** |
| **Total:** | | **166** |
